# Validation of galectin-1 as potential diagnostic biomarker of early rheumatoid arthritis

**DOI:** 10.1038/s41598-020-74185-8

**Published:** 2020-10-20

**Authors:** Ana Triguero-Martínez, Hortensia de la Fuente, Nuria Montes, Ana María Ortiz, Emilia Roy-Vallejo, Santos Castañeda, Isidoro González-Alvaro, Amalia Lamana

**Affiliations:** 1 Rheumatology Department, Hospital Universitario La Princesa, Instituto de Investigación Sanitaria La Princesa (IIS-IP), Madrid, Spain; 2 Immunology Department, Hospital Universitario La Princesa, Instituto de Investigación Sanitaria La Princesa (IIS-IP), Madrid, Spain; 3 Internal Medicine Service, Hospital Universitario La Princesa, Instituto de Investigación Sanitaria La Princesa (IIS-IP), Madrid, Spain; 4 Cell Biology Department, Facultad de Biología, Universidad Complutense de Madrid, Madrid, Spain

**Keywords:** Biomarkers, Rheumatology

## Abstract

Galectin 1 (Gal1) is a lectin with a wide cellular expression that functions as a negative regulator of the immune system in several animal models of autoimmune diseases. Identification of patients with rheumatoid arthritis (RA) has improved during the last decade, although there is still a need for biomarkers allowing an early diagnosis. In this regard, it has been recently proposed that Gal1 serum levels are increased in patients with RA compared to the general population. However, this topic is controversial in the literature. In this work, we provide additional information about the potential usefulness of Gal1 serum levels as a biomarker for RA diagnosis. We studied Gal1 serum and synovial fluid levels and clinical parameters in samples from 62 patients with early arthritis belonging to the PEARL study. In addition, 24 healthy donors were studied. We found that both patients fulfilling RA criteria and patients with undifferentiated arthritis showed higher Gal1 levels than healthy donors. Similar findings were observed in synovial fluid, which showed even higher levels than serum. However, we did not find correlation between Gal1 levels and disease activity or disability. Therefore, our results suggest that Gal1 could be a diagnostic but not a severity biomarker.

## Introduction

Rheumatoid arthritis (RA) is the most common autoimmune rheumatic disease. Although the ethiopathogenesis of RA has not been fully elucidated, it is considered a complex disease in which the interaction between genetic, environmental and stochastic factors leads to a chronic infiltration of the synovial membrane by a variety of cells of the immune system. These infiltrated cells cause chronic production of proinflammatory cytokines (TNF-α, IL-1β and IL-6) and metalloproteinases that usually leads to joint destruction due to cartilage degradation and bone erosion^1^.

During the last years, solid evidence has confirmed the “window of opportunity” concept, which establishes the possibility of reaching better outcomes with early treatment with disease modifying anti-rheumatic drugs (DMARDs)^2,3^. On the other hand, a better knowledge of the regulation of the immune system can improve our ability to tailor treatment intensity. At present, it is accepted for seropositive RA that the interaction between HLA DRB1 alleles encoding the “shared epitope” and smoking habit increases the risk of developing autoreactive T and B clones. These autoreactive clones in turn produce antibodies against citrullinated proteins (ACPA), which lead to chronic synovitis^4^. However, more than 100 loci have been suggested to contribute to the genetic risk of RA development^5^ and several environmental factors^4^ may also interact with them to promote RA, likely leading to a very heterogeneous clinical course.

Although some experts have suggested to treat intensively arthritis to abrogate inflammation^6^, it seems wise to avoid over-treating patients less likely to develop a severe disease^7^. In this regard, the landscape can be even more complex, since we have recently described that genetic variants in the region encoding for vasoactive intestinal peptide (VIP), which have not been associated with the risk of developing RA, contribute to the heterogeneity of disease severity^8^. Therefore, the ability to predict the heterogeneous clinical spectrum of RA has improved with the introduction of ACPA^9^, although it is still low due to our limitations in understanding its molecular complexity.

Galectins are lectins that bind β-galactoside carbohydrates^10,11^ via carbohydrate recognition domains (CRDs). At least 15 mammalian galectins have been described, all containing 1 or 2 CRDs of approximately 130 amino acids. The variety of binding partners and the wide distribution of galectins allow them to participate in multiple biological functions, including the regulation of the immune system^12^.

Galectin 1 (Gal1) is a protein of approximately 14 KDa that is able to form homodimers. It is highly expressed in immune cells and its expression is modulated during activation and differentiation^12^. Gal1 plays an immunosuppressive and anti-inflammatory role due to its pro-apoptotic effect in activated lymphocytes^13^, promoting the polarization of Th1 responses towards Th2^14^ and Th17 towards Treg^15^, as well as blocking the in vitro secretion of pro-inflammatory cytokines (IL-2, IFNγ and TNFα)^16,17^. In addition, treatment with recombinant Gal1 improves clinical manifestations of established collagen-induced arthritis (CIA)^15^. A recent cross-sectional study described that RA patients show higher Gal1 serum levels^18^. However, other authors have described similar Gal1 serum levels in RA patients and healthy controls^19^.

Additional controversial data have been reported describing reduced expression of Gal1 in synovial tissue of patients with juvenile idiopathic arthritis^20^, absence of Gal1 expression at cartilage invasion sites in RA patients^21^ or decreased Gal1 levels in synovial fluid in comparison to healthy controls^19^.

Therefore, the role of Gal1 as a biomarker for RA remains controversial. In this work, we aim to describe the evolution of Gal1 serum level in a longitudinal study in patients with early arthritis (EA), as well as to compare Gal1 levels in synovial fluid from patients with different inflammatory arthritis and osteoarthritis.

## Results

### Study population

In order to validate the report by Mendez-Huergo et al. describing that patients with RA show higher Gal1 serum levels than healthy donors^18^, we measured Gal1 levels in serum from 62 patients included in the PEARL study. Patients were clustered in two different populations (Pop1 and Pop2) and 24 healthy donors (HD) were also studied. The characteristics of the 3 populations are shown in Table 1. No significant differences were found between populations. The only difference was that Pop1 included 30 RA patients, while Pop2 was a mixed population with a 34% of undifferentiated arthritis (UA); therefore, a significantly higher baseline DAS28 was observed in Pop1.

**Table 1:** Baseline characteristics of the populations studied.

	Population 1	Population 2	Healthy donors	p
	(n = 30)	(n = 32)	(n = 24)	
Female; n (%)	25 (83.33)	27 (84.38)	20 (83.33)	1
Age; p50 [p25–p75]	52 [39–70]	52 [43 – 60]	52 [36–64]	0.79
BMI; p50 [p25–p75]	26.46 [23.5–29.27]	26.78 [22.87–30.26]	28.23 [25.05–29.37]	0.646
RA/UA; n (%)	30 (100)/0 (0)	21 (65.63)/11 (34.38)		< 0.001
Disease duration (months); p50 [p25–p75]	6.48 [2.76–8.83]	2.78 [2–7.81]		0.060
RF positive; n (%)	22 (73.33)	16(50)		0.059
ACPA positive; n (%)	20(66.67)	16(50)		0.184
DAS28; p50 [p25–p75]	5.12 [3.89–5.8]	3.91 [2.98–6.34]		0.049
HAQ; p50 [p25–p75]	1.06 [0.62–1.62]	1 [0.81–1.37]		0.970

p50: median; p25-p75: interquartile range; BMI: body mass index; RA: rheumatoid arthritis; UA: undifferentiated arthritis; RF: rheumatoid factor; ACPA: anti-citrullinated protein antibodies; DAS28: disease activity score estimated with 28 joint count. HAQ: health assessment questionnaire.

### Early arthritis patients show higher Gal1 serum levels than healthy controls

**Figure 1:**
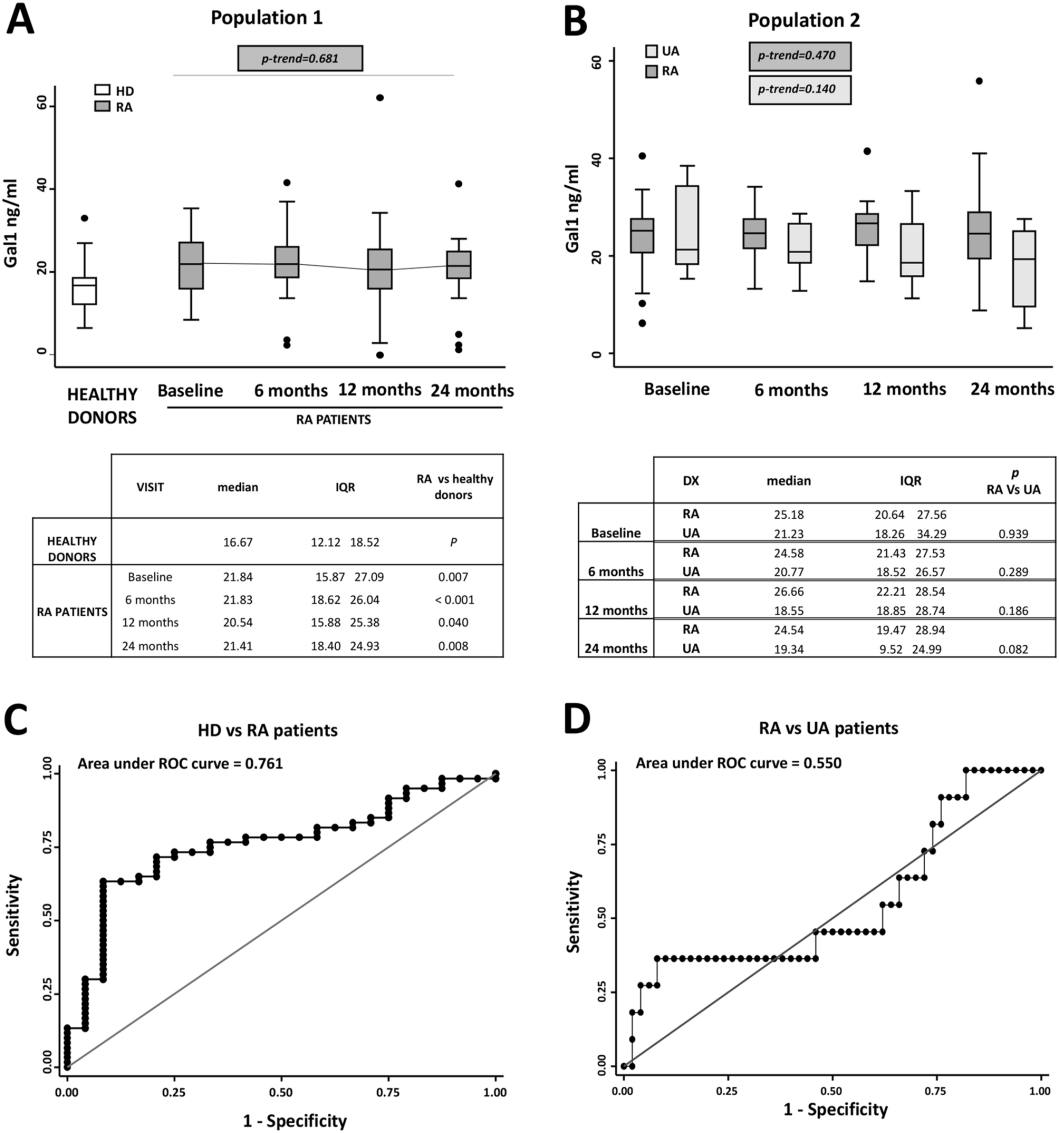
Gal1 serum levels are increased in early arthritis patients compared with healthy donors. (**A**) Determination of Gal1 serum levels by ELISA in healthy donors (HD) and rheumatoid arthritis (RA) patients from PEARL study (Population 1). (**B**) Determination of Gal1 serum levels by ELISA in early arthritis patients (undifferentiated arthritis [UA] and RA) from PEARL study (Population 2). Data are shown as interquartile range (p75 upper edge of box, p25 lower edge, p50 midline) as well as the p95 (line above box) and p5 (line below). Dots represent outliers. Statistical significance for the trend of Gal1 across the visits in patients was determined with the Cuzick’s non-parametric test. Significance threshold was set at p-trend < 0.05. Tables below the panels show the statistical significance between HD and each visit of RA patients (**A**) or between RA and UA patients in each visit (**B**). Statistical significance was determined with Mann–Whitney test. Significance threshold was set at p < 0.012 due to multiple comparisons, according to Bonferroni correction. (**C**) ROC curve analysis to assess Gal1 capacity to discriminate between RA patients and healthy donors. (**D**) ROC curve analysis to assess Gal1 capacity to discriminate between RA patients and UA patients.

As it is shown in Fig. 1A, baseline Gal1 serum levels in RA patients of Pop1 were significantly higher than levels of healthy controls. Furthermore, this increased expression was maintained all along the follow-up and samples from all 4 visits of patients showed significantly higher Gal1 levels than samples from healthy controls (baseline p = 0.007; 6 months p < 0.001; 12 months p = 0.040; and 24 months p = 0.008; Fig. 1A). Furthermore, a Gal1 serum concentration above 19.12 ng/ml could differentiate RA patients from controls (AUC = 0.761) with 71% sensitivity and 79% specificity (Fig. 1C; LR+ 3.44 and LR− 0.35).

In addition, we were interested in whether the evolution of Gal1 levels was different in RA patients versus those with UA. Our data showed no significant differences between the baseline expression levels of RA patients with respect to UA, with a poor capability to differentiate between RA and UA (AUC = 0.55), being the best cut-off point 34 ng/ml with a 27% sensitivity and 96% specificity (LR+ 6.81, LR− 0.76; Fig. 1D). Although no significant down regulation of Gal1 was observed throughout the follow-up either in RA or in UA, a slightly higher Gal1 was observed in serum from patients with RA at the 2-year visit (p = 0.08, Fig. 1B).

### Gal1 serum levels are not associated with activity or disability in EA patients

**Figure 2:**
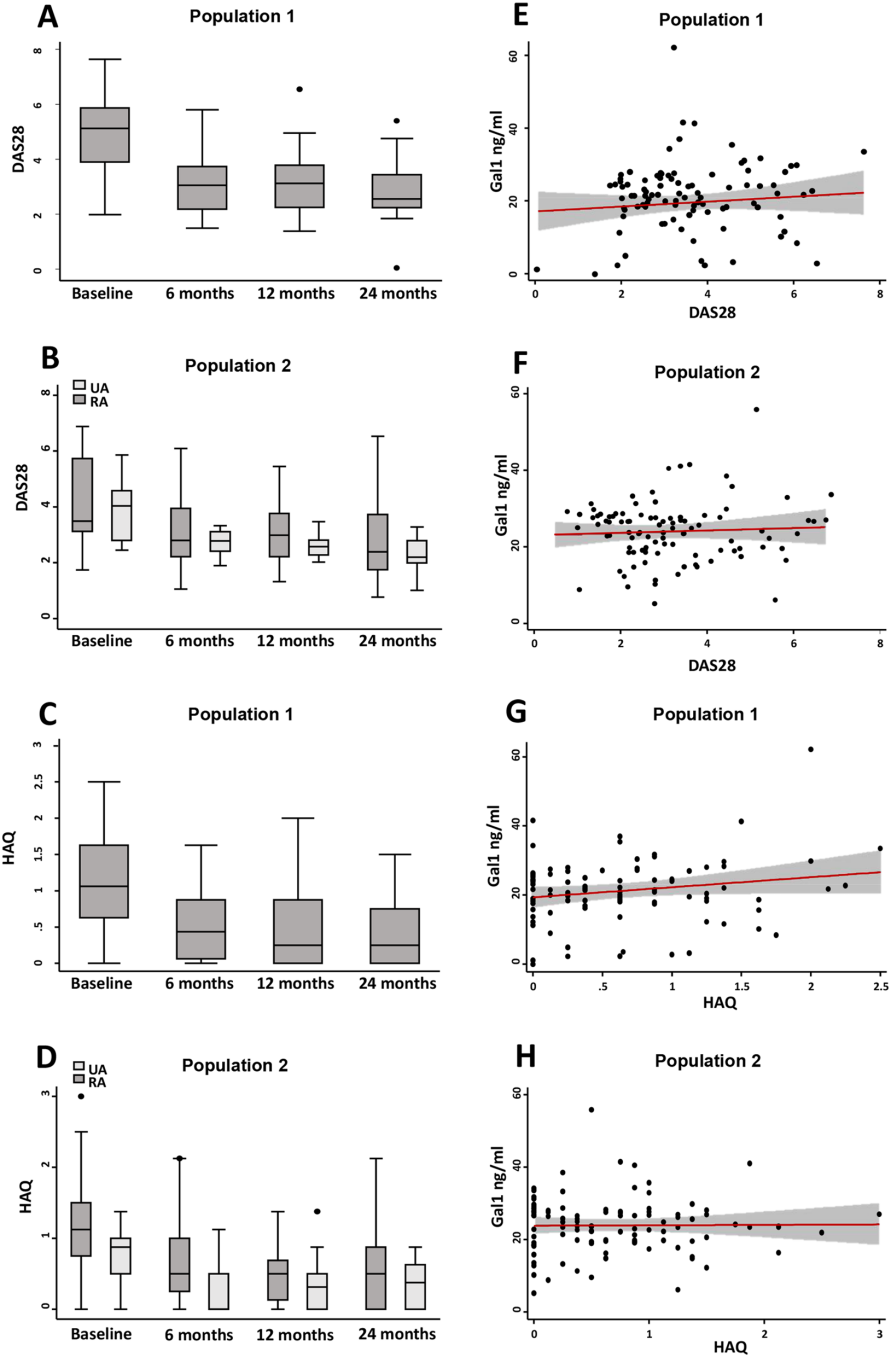
Gal1 serum levels do not correlate with clinical severity parameters in early arthritis. Evolution along the follow-up of disease activity assessed by DAS28 (A Population 1; B Population 2) and disability assessed by HAQ (C Population 1; D Population 2) in early arthritis. Absence of correlation of Gal1 serum levels with DAS28 (E Population 1; F Population 2) and HAQ (G Population 1; H Population 2) in early arthritis. In (**A**–**D**), data are shown as interquartile range (p75 upper edge, p25 lower edge, p50 midline), p95 (line above the box) and p5 (line below the box). Dots represent outliers. In (**E**–**H**), data are shown as dot-plots and their fitted linear prediction with 95% confidence interval (grey shadow) estimated using the *twoway* command of Stata with the *lfitci* option. Pearson correlation test was used to determine the level of significance.

To determine whether Gal1 serum levels could be a biomarker of severity, we studied their correlation with either disease activity assessed by DAS28 or disability measured by HAQ score. As it is shown in Fig. 2A,B, disease activity decreased along the follow-up in both Pop1 (Fig. 2A) and Pop2 (Fig. 2B) in response to treatment (Table S2). However, Gal1 levels remained elevated without significant fluctuations throughout the follow-up (Fig. 1A,B). Therefore, no significant correlation was observed between DAS28 and Gal1 serum levels in both populations (Fig. 2E,F). A similar finding was observed with disability, HAQ score improved throughout the follow-up (Fig. 2C,D), whereas no significant correlation was observed between HAQ and Gal1 serum levels (Fig. 2G,H).

Also, we performed a multivariate analysis to determine which variables could be associated with Gal1 serum levels. We observed that age and female gender positively correlated with Gal1 levels (Table 2), while, as mentioned above, time of frozen storage negatively correlated in a very significant way (Table 2 and Fig. S1). After adjustment for these confounding variables, the analysis confirmed that neither DAS28 nor HAQ (Table 2, upper and lower sections of the table, respectively) correlated with Gal1 serum levels throughout the follow-up.

**Table 2:** Relationship between Gal1 (ng/ml) serum levels and disease activity or disability.

	Disease activity	
	β Coeff. (95 % CI)	p value
DAS28	0.51 (− 0.32 to 1.36)	0.231
Female gender	2.15 (− 1.5 to 5.8)	0.256
**Age (years)**		
< 45	Reference	–
45–65	4.68 (1.40 to 7.95)	0.005
> 65	2.87 (− 0.64 to 6.39)	0.109
Time of frozen storage (months)	− 0.03 (− 0.06 to − 0.01)	0.002

	Disability	
	β Coeff. (95 % CI)	p value
HAQ	1.26 (− 0.62 to 3.15)	0.189
Female gender	1.87 (− 1.93 to 5.67)	0.336
**Age (years)**		
< 45	Reference	–
45–65	4.55 (1.29 to 7.81)	0.006
> 65	2.54 (− 1.06 to 6.14)	0.167
Time of frozen storage (months)	− 0.03 (− 0.06 to − 0.01)	0.005

DAS28, 28-Joint Disease Activity Score; HAQ, Health Assesment Questionnaire; Coeff. Coefficient.

### Gal1 serum levels correlate with serum IL6 in RA

**Figure 3:**
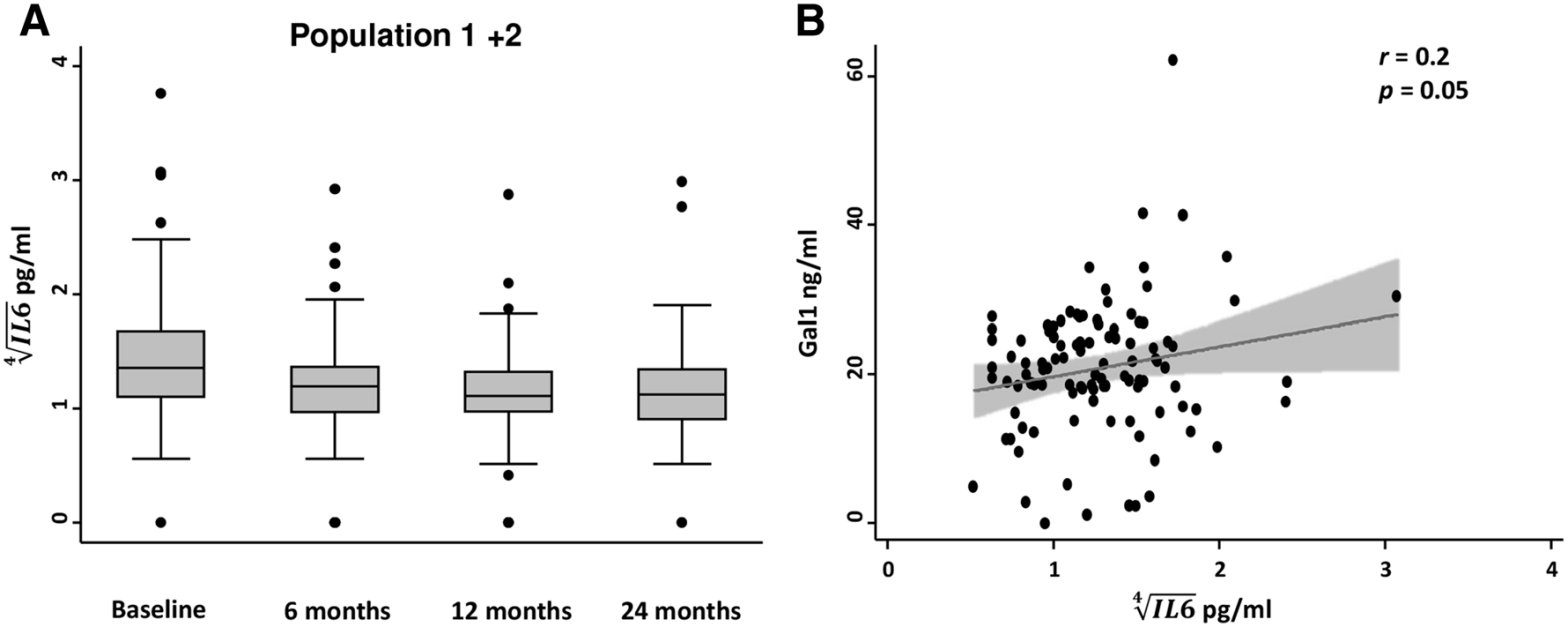
Gal1 serum levels correlate with IL-6 serum levels in early arthritis. (**A**) Evolution along the follow-up of IL6 serum levels (Population 1 plus Population 2) in early arthritis patients. Data are shown as interquartile range (p75 upper edge, p25 lower edge, p50 midline), p95 (line above the box) and p5 (line below the box).Dots represent outliers. (**B**) Moderate correlation between Gal1 and IL6 serum levels (Population 1 plus Population 2) in early arthritis patients. Data are shown as dot-plots and their fitted linear prediction with 95% confidence interval (grey shadow) estimated using the *twoway* command of Stata with the *lfitci* option. Pearson correlation test was used to determine the level of significance.

Since it has been described that Gal1 is able to decrease IL6 production in vitro^22^, we were interested in determining whether Gal1 was associated with the serum levels of the proinflammatory cytokine IL6 in our EA patients. As observed above for disease activity and disability, IL6 levels decreased along the follow-up (Fig. 3A). Nevertheless, we observed a moderate, but significant correlation between Gal1 serum levels and IL6 levels (r = 0.2, p = 0.05; Fig. 3B). To confirm that this association was not due to some bias, we performed a multivariate analysis in which, as expected, we observed that IL6 serum levels positively correlated with levels of disease activity and with increasing age. Conversely, methotrexate treatment was associated with decreased IL6 levels (Table 3), as previously described^23,24^. After adjustment for these variables a significant association between Gal1 and IL6 serum levels was still observed (Table 3) confirming the correlation showed in Fig. 3B.

**Table 3:** Relationship between IL6 (pg/ml) and Gal1 (ng/ml) serum levels.

	β Coeff. (95 % CI)	p value
Galectin1 (ng/ml)	0.008 (0.001 to 0.01)	0.023
**Age (years)**		
< 45	Reference	–
45–65	0.18 (− 0.06 to 0.43)	0.154
> 65	0.17 (− 0.06 to 0.40)	0.162
**DAS28 **		
Remission	Reference	–
Low DA	0.09 (− 0.09 to 0.27)	0.327
Moderate DA	0.31 (0.15 to 0.47)	< 0.001
High DA	0.41 (0.18 to 0.64)	< 0.001
Methotrexate yes/no	− 0.16 (− 0.31 to − 0.004)	0.044

DAS28: disease activity score estimated with 28 joint count; Coeff., coefficient; IL6, interlukin 6; DA: disease activity.

### Gal1 synovial fluid levels are increased in RA patients

Finally, we were interested in determining whether Gal1 was also locally increased in patients with RA. Thus, we studied Gal1 levels in SF from 20 patients with RA, 20 with osteoarthritis and 22 with microcrystalline arthritis (gouty and chondrocalcinosis arthritis).

**Figure 4:**
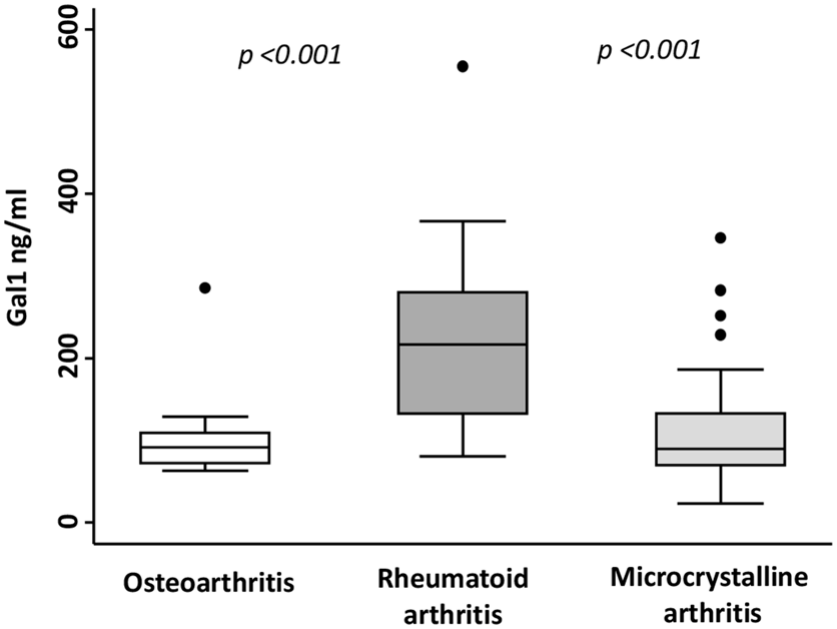
Gal1 synovial fluid levels are increased in RA patients. Determination of Gal1 synovial fluid levels by ELISA in OA, microcrystalline arthritis and RA patients. Data are shown as interquartile range (p75 upper edge of box, p25 lower edge, p50 midline) as well as the p95 (line above box) and p5 (line below). Dots represent outliers. Statistical significance was determined with ANOVA. Significance threshold was set at p < 0.05.

We observed that RA patients had significantly higher Gal1 levels than OA patients (p < 0.001, Fig. 4) and patients with microcrystalline arthritis (p < 0.001, Fig. 4). Furthermore, in six RA patients, not included either in pop1 or in pop2, we had the opportunity to obtain in parallel SF and blood samples and the levels of Gal1 in SF were significantly higher than those detected in serum, (p < 0.001, Fig. S2).

## Discussion

The main findings of this work are: (i) the validation of serum Gal1 measurement as a possible diagnostic biomarker for RA; and (ii) the presence of even higher levels of this molecule in synovial fluid from patients with RA, as well as in comparison with synovial fluid from other mechanic or inflammatory processes such as osteoarthritis and microcrystalline arthritis.

The current paradigm of RA management is to achieve remission as soon as possible^25^. However, despite the new classification criteria for RA including rheumatoid factor and anti citrullinated protein antibodies as diagnostic biomarkers^26^, many patients at early arthritis clinics do not fulfil these criteria. Many efforts have been made to improve the accuracy of these classification criteria, especially including new autoantibodies, such as anti carbamylated protein antibodies^27^.

In this regard, until now, the available data on Gal1 serum levels in RA patients and their possible correlation with clinical severity parameters were controversial. The first study comparing Gal1 serum levels from RA patients and HD found no differences between them^19^. On the other hand, the recent article by Mendez-Huergo et al. described that Gal1 serum levels were higher in RA patients than in HD and correlated with clinical severity parameters^18^. Our study supports the later and, additionally, it has the advantage that it was performed in patients with early arthritis using a longitudinal design. This design allowed to analyse Gal1 levels at the beginning of the disease in untreated patients (visit 1), as well as during their follow-up after the establishment of the treatment. A baseline Gal1 serum level > 19 ng/ml showed a good performance to discriminate HD from either RA or UA patients. The difference between the cut-off point proposed in the study by Mendez-Huergo et al. and our study could be due to the use of different ELISA kits to measure Gal1 in serum^18^. In our case, we used a commercial kit, which showed very low variability between tests. The use of standardized ELISA kits instead of home-made kits would be a good strategy since a fundamental point in the search for biomarkers and their subsequent validations is reproducibility.

The other point of controversy is whether Gal-1 levels may be a severity biomarker or could be considered as an acute phase reactant. The only work that tried to address this point was that of Mendez-Huergo et al., which only found a mild correlation with parameters of disease activity^18^. Our work did not show association with disease activity or disability. Despite these outcomes improved with treatment along the follow-up, no significant decrease in Gal-1 serum levels was observed. Furthermore, no association with severity outcomes, such as achievement of remission, radiological progression, cumulative intensity of treatment or need for biological therapy was detected. Additional studies with sera from different inflammatory arthropathies would be necessary to determine whether increased serum levels of Gal-1 are RA specific and can be used as a diagnostic biomarker.

To gain further insight into the potential diagnostic utility of Gal1, we measured its expression in SF from different pathologies. Previously, Xibille-Friedmann et al. observed that the level of Gal1 was significantly lower in RA patients than in patients with non-arthritic knee effusion^19^. On the contrary, our data show that Gal1 synovial levels in RA are higher compared to osteoarthritis and microcrystalline arthritis. These different results could be explained by the treatment of SF samples with hyaluronidase in the work of Xibille-Friedmann et al.^19^, which could have interfered with their measurement of Gal1 in SF. In addition, they did not take into account the time of frozen storage of the samples, which is a variable that can significantly affect Gal1 detection, as we have demonstrated here. Finally, in their study, low levels of Gal1 correlated with the presence of anti-Gal1 antibodies in the joint^19^, suggesting that a technical interference between the antibodies of their ELISA kit and the possible presence of anti-Gal1 antibodies may have occurred in their experiments. In addition, our study supports a local production of Gal1 in RA patients, since its levels were significantly higher in SF than in serum.

Regarding pathogenesis of the disease, IL6 plays an important role in RA, which has allowed the development of IL-6 signalling blockade as an effective tool in the treatment of RA patients^1^. Treatment with Gal1 reduces proinflammatory cytokine levels in vitro^16,17^; however, its effect on IL6 production has not been described. Although IL6 serum levels decreased along the follow-up while Gal1 levels mostly remained unaffected, we were still able to detect a moderate association between them. Therefore, it is likely that Gal1 and IL6 are related in the etiopathogenesis of the RA, at least in a subpopulation of patients. In this regard, the slight differences observed in Gal1 serum levels between patients with RA and UA at the last visit of follow-up could be due to the heterogeneity of the UA population. Thus, patients not fulfilling RA criteria because they are efficiently treated at very early stages of the disease could behave as RA patients and still display high Gal1 at this last visit, whilst patients with other disorders may undergo Gal1 down-regulation once disease activity is controlled. Another explanation could be the low number of UA patients included in the study would not allow the Cuzick’s test to detect a significant trend in Gal1 levels along the follow-up. However, we believe that it would be interesting to continue studying differences in Gal1 serum levels between RA and UA patients, since Gal1 levels could be useful for early diagnosis in EA patients. It would also be interesting to unravel the combined role of Gal1 and IL6 in the pathophisiolgy of RA.

In conclusion, our work suggests that Gal1 serum levels may have role as a diagnostic biomarker in patients with early RA, although they cannot be considered a severity biomarker. In addition, they support that Gal1 and IL6 may be related during RA etiopathogenesis. Therefore, our study opens a door to further characterise the role of Gal1 in the pathogenic basis of RA, especially by exploring its relationship with IL6.

## Materials and methods

### Serum sample from early arthritis population

The present study was performed with data and samples from the Princesa Early Arthritis Register Longitudinal (PEARL) study carried out at the Hospital Universitario de La Princesa, Madrid, Spain. The PEARL study comprises patients with one or more swollen joint and symptoms with ≤ 1 year of evolution. The register protocol includes 4 visits during a 2-year follow-up (0, 6, 12 and 24 months). Socio-demographic, clinical, therapeutic and laboratory data are recorded and included in an electronic database. Biological samples are collected at each visit and stored at − 80 °C in the Instituto de Investigación Sanitaria La Princesa (IIS-IP) Biobank for translational research. More detailed description of PEARL protocol has been previously published^28^.

At the end of follow-up,all EA patients are classified as RA if they fulfil 1987 ACR classification criteria^29^ or as UA as described by Verpoort et al.^30^. Patients with other defined diagnosis (connective tissue diseases, psoriatic arthritis, gout or osteoarthritis) are excluded from the study.

For this study, two independent PEARL populations of patients were studied. Population 1 (Pop1) comprised 30 RA patients with data from 95 visits (average 3.2 per patient) and Population 2 (Pop2) (validation group) comprising 21 RA and 11 UA patients with data from 107 visits (average 3.2 per patient). Twenty-four healthy donors were also analysed. Clinical characteristics of all populations are shown in Table 1.

### Synovial fluid sample population

Synovial fluid (SF) samples were obtained from therapeutic or diagnostic arthrocentesis after oral informed consent registered in the clinical chart. Those samples contaminated with blood were discarded. Samples were centrifuged at 2000 rpm during 20 min at room temperature and the cell-free supernatants were collected and stored at − 80 °C until analysis. The samples were anonymised and only information about diagnosis was collected. For this study, samples from 20 RA patients, 20 osteoarthritis (OA) patients and 22 microcrystalline arthritis patients (gouty and chondrocalcinosis arthritis) were analysed.

### Measure of serum and synovial fluid Gal1

Gal1 serum and SF levels were measured using Quantikine Human Gal1 Immunoassay (R&D Systems, Minneapolis, USA). The procedure was performed according to the manufacturer's instructions. Absorbance was measured in a spectrophotometer (Innogenetics Diagnostica y terapeutica S.A.U, Barcelona, Spain) at 450 nm with correction at 620 nm. Measurements for all samples were performed in triplicate.

### Time of frozen storage of the samples

Since time of frozen storage of the samples analysed ranged from 6 to 200 months, we tested whether this circumstance could affect Gal1 detection. As Supplementary Fig. S1 shows, time of frozen storage significantly affected the capability to detect Gal1 (p = 1.47 10^–5^ and R^2^ = 0.07), although differences between healthy donors and patients were clearly observed even in the samples with longer frozen storage. Therefore, the variable time of frozen storage was used as a confounding variable in the following multivariate analyses.

### Statistical analysis

Statistical analyses were performed using Stata 14 for Windows (Stata Corp LP, College Station, TX, USA). Most quantitative variables followed a non-normal distribution, so they were represented as median and interquartile range (IQR) and the Mann Whitney or Kruskal–Wallis tests were used to analyse significant differences. When a variable was normalised (see below) the Student t-test was applied. Qualitative variables were described using a calculation of the proportions and the χ^2^ or Fisher’s exact test was used to compare categorical variables. The Pearson correlation test was applied to analyse correlation between quantitative variables.

The Cuzick’s test, an extension of the Wilcoxon rank-sum test, was used to determine statistical significance of the distribution trend across ordered groups in variables such as visits or level of disease activity.

The relationship between time of frozen storage (days) and Gal1 serum levels was analysed by polynomial second degree regression conducted with R version 3.5.0^31^.

### Multivariate analyses

In order to identify those variables associated with variation in Gal1 serum levels we fitted a multivariate analysis using generalised linear models nested by patient and visits using the *xtgee* command of STATA. The population-averaged generalised estimating equations were first modelled by adding all variables with a *p* value < 0.15 to the bivariate analysis. The final models were constructed using quasi-likelihood estimation based on the independence model information criterion^32^ and Wald tests, removing all variables with *p* > 0.15. The variable time of frozen storage was always included in the multivariate analysis. Once the best model was obtained, several variables related with assessment of RA were forced in the model in order to determine whether there was an association with Gal1 or not.

In addition, to determine whether there was an association between Gal1 and IL6 serum levels, we fitted another multivariate analysis using xtgee command of Stata as described above, but in this case the dependent variable was IL6 serum levels, which did not follow a normal distribution, so the variable was transformed through double square root transformation. The best model was obtained as described above.

In order to assess the ability of Gal1 serum levels to discriminate between RA patients and healthy donors or between RA and UA patients we generated Receiver Operating Characteristic (ROC) curves through the command roctab and the option *graph*. Each cut-off point was selected on the basis of the best trade-off values between sensitivity, specificity, cases correctly classified and positive (LR+) and negative (LR−) likelihood ratios reported using the command roctab with the option detail.

### Ethical considerations

PEARL study is conducted according to the principles expressed in the Helsinki Declaration of 1983 and it was approved by the Research Ethics Committee of Hospital Universitario de La Princesa (PI-518; March 28th, 2011). All patients included were over 18 years old and signed a written informed consent at study entry and samples and data from patients included in this study were provided by the IIS-IP Biobank (ISCIII B.0000763). All samples were processed following standard operating procedures with the appropriate approval of the Ethics and Scientific Committees.

## Supplementary information

Supplementary Information 1.

Supplementary Information 2.

Supplementary Information 3.

## Data Availability

All raw data from serum and synovial fluid required to reproduce our results are provided as the Supplementary files “serum.xlsx” and “synovial.xlsx”. Any issue regarding the interpretation of the variables, please contact with Dr. Isidoro González-Álvaro: isidoro.ga@ser.es
