# Supplementary material for: Validation of galectin-1 as potential diagnostic biomarker of early rheumatoid arthritis
Source: Sci Rep. 2020 Oct 20;10:17799. doi: 10.1038/s41598-020-74185-8 (PMC7576119; doi:10.1038/s41598-020-74185-8)
Supplement: Supplementary file 1 — Supplementary Information 1. [file 41598_2020_74185_MOESM1_ESM.docx]

**Validation of Galectin-1 as potential diagnostic biomarker of early rheumatoid arthritis.**

Ana Triguero-Martínez^1^, [ana6n92@gmail.com](mailto:ana6n92@gmail.com)

Hortensia de la Fuente^2^, [hortensiadelafuente@gmail.com](mailto:hortensiadelafuente@gmail.com)

Nuria Montes^1^, [nuria.montes.casado@gmail.com](mailto:nuria.montes.casado@gmail.com)

Ana María Ortiz^1^, [lanult@yahoo.es](mailto:lanult@yahoo.es)

Emilia Roy-Vallejo^3^, [eroyvallejo@gmail.com](mailto:eroyvallejo@gmail.com)

Santos Castañeda^1^, [scastas@gmail.com](mailto:scastas@gmail.com)

Isidoro González-Alvaro^1^*, [isidoro.ga@ser.es](mailto:isidoro.ga@ser.es) Phone: 34-915202438. Fax: 34-915202374.

Amalia Lamana^4^*, [amaliala@ucm.es](mailto:amaliala@ucm.es) Phone: 34-915202438. Fax: 34-915202374.

^1^Rheumatology Department, Hospital Universitario La Princesa, Instituto de Investigación Sanitaria La Princesa (IIS-IP), Madrid. Spain.

^2^Immunology Department, Hospital Universitario La Princesa, Instituto de Investigación Sanitaria La Princesa (IIS-IP), Madrid. Spain.

^3^Internal Medicine Service, Hospital Universitario La Princesa, Instituto de Investigación Sanitaria La Princesa (IIS-IP), Madrid. Spain

^4^Cell Biology Department, Facultad de Biología, Universidad Complutense de Madrid, Madrid, Spain.

* These authors share corresponding authorship

SUPPLEMENTARY MATERIAL

Supplementaray Figures.


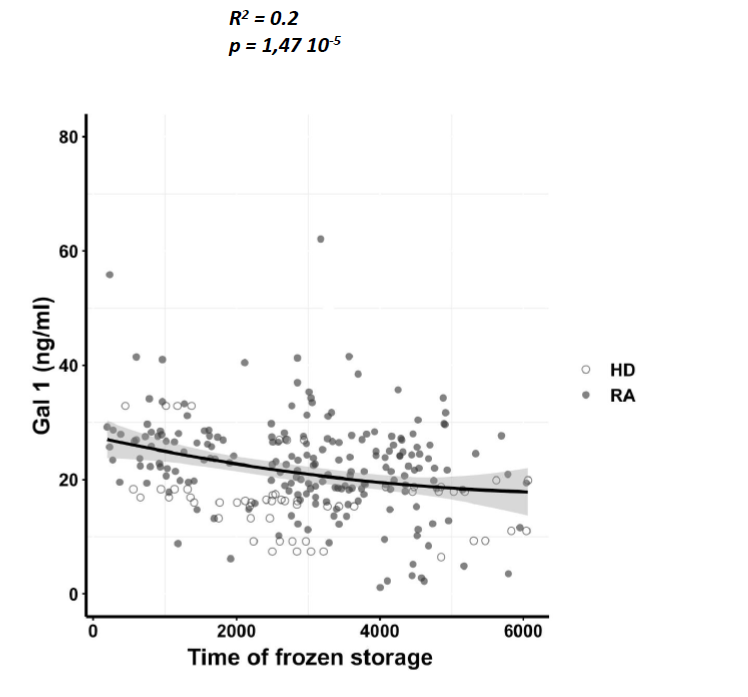


**Supplementary figure 1**. Gal1 serum levels decrease with time of frozen storage. Pairwise polynomial second-degree regression between time of frozen storage (days) and Gal1 serum levels (ng/mL) in healthy donors (HD) and rheumatoid arthritis (RA) patients. Data are shown as dot-plots and their fitted linear prediction with 95% confidence interval (grey shadow).


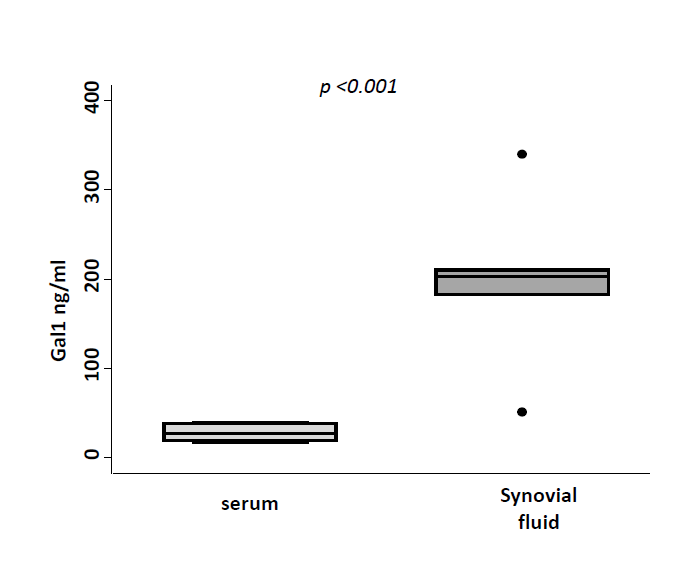


**Supplementary figure 2.** Gal1 synovial fluid levels are higher than Gal1 serum levels in RA patients. Determination of Gal1 synovial fluid and serum levels by ELISA in RA patients. Data are shown for Gal1 levels as interquartile range (p75 upper edge of box, p25 lower edge, p50 midline) as well as the p95 (line above box) and p5 (line below). Dots represent outliers. Statistical significance was determined with t-test. Significance threshold was set at p <0.05.

Supplementary tables

**Supplementary Table 1. Other variables at baseline.**

|  | **Population 1** | **Population 2** | **p** |
| --- | --- | --- | --- |
|  | (n = 30) | (n = 32) |  |
| CRP; p50 [p25-p75] | 1.1 [0.2 – 2.9] | 0.7 [0.2 – 3] | 0.589 |
| IL6 ; p50 [p25-p75] | 6.77 [3.1 – 8.77] | 4.49 [1.92 – 12.01] | 0.730 |
| NAD ; p50 [p25-p75] | 4 [1 – 9] | 3 [0.5 – 12] | 0.937 |
| NAT ; p50 [p25-p75] | 6 [3 – 9] | 4 [2.5 – 7] | 0.282 |
| n: number; p50: median or percentile 50; p25-p75: range between percentiles 25 and 75 or interquartile range; CRP, C-reactive protein; IL6, interlukin 6; NAD, tender joint count (0-28); NAT, swollen joint count (0-28)  **Supplementary Table 2. Treatment in both populations during the follow-up**   \|  \| **POPULATION 1** \| \| \| **POPULATION 2** \| \| \| \| --- \| --- \| --- \| --- \| --- \| --- \| --- \| \|  \| **6 months** \| **12 months** \| **24 months** \| **6**  **months** \| **12 months** \| **24 months** \| \| Methotrexate (n) \| 22 \| 16 \| 18 \| 23 \| 24 \| 22 \| \| Leflunomide (n) \| 6 \| 6 \| 4 \| 2 \| 4 \| 9 \| \| Hidroxicloroquine (n) \| 8 \| 8 \| 8 \| 4 \| 4 \| 4 \| \| Salzopyrine (n) \| 1 \| 2 \| 1 \| 3 \| 3 \| 3 \| | | | |
